# Development of a Loop Mediated Isothermal Amplification for Diagnosis of* Ascaris lumbricoides* in Fecal Samples

**DOI:** 10.1155/2016/7376207

**Published:** 2016-11-01

**Authors:** Esther A. Shiraho, Agola L. Eric, Ibrahim N. Mwangi, Geoffrey M. Maina, Joseph M. Kinuthia, Martin W. Mutuku, Robert M. Mugambi, Jackson M. Mwandi, Gerald M. Mkoji

**Affiliations:** ^1^Institute of Tropical Medicine and Infectious Diseases, Jomo Kenyatta University of Agriculture and Technology, P.O. Box 62000-00200, Nairobi, Kenya; ^2^Centre for Biotechnology Research and Development, Kenya Medical Research Institute (KEMRI), P.O. Box 54840-00200 Nairobi, Kenya; ^3^Vector Borne Disease Control Unit Laboratory, Msambweni County Referral Hospital, P.O. Box 8, Msambweni, Kenya

## Abstract

*Ascaris lumbricoides* is a nematode parasite that causes the common tropical infection ascariasis in humans. It is also considered among the neglected tropical diseases. Diagnosis relies mainly on microscopy-based methods which are laborious, are limited by low sensitivity, and require high expertise. We have developed a loop mediated isothermal amplification (LAMP) for diagnosis of ascariasis in fecal samples, based on the first internal transcribed (ITS-1) spacer region of the ribosomal DNA. We used Primer Explorer V4 software to design primers.* Ascaris* adult and ova were obtained from naturally infected school children, whose parents/guardians gave consent for their participation in the study. Genomic DNA was extracted using alkaline lysis method and amplified by LAMP at 63°C for 45 minutes. LAMP products were visualized by naked eyes after adding SYBR Green dye and also on agarose gel. LAMP successfully and reliably detected* Ascaris* DNA from a single egg and in fecal samples. The assay specifically detected* Ascaris* DNA without amplifying DNA from ova of other parasites which commonly coexist with* A. lumbricoides* in feces. The developed LAMP assay has great potential for use in ascariasis diagnosis at the point of care and in low infection intensity situation that characterize control and elimination campaigns.

## 1. Introduction

*Ascaris lumbricoides* causes soil-transmitted helminthiases (STHs) worldwide and is a major public health problem, considered as one of the neglected tropical diseases, and currently targeted for elimination [1]. It is the causal agent of ascariasis with an estimated global prevalence of 1.2 billion people [2], majority of whom are children living in sub-Saharan Africa [3]. Adults are also often infected with* A. lumbricoides* but prevalence and infection intensities tend to be much lower than in children [4]. The epidemiological factors responsible for ascariasis and other soil-transmitted helminth infections include poor sanitation and hygiene, inadequate water supplies, and poor health care systems [5].

Infection with ascariasis is through the fecal-oral route [6]. Diagnosis is mainly by examining fecal sample under a microscope for the parasite ova using the Kato-Katz technique as recommended by World Health Organization (WHO) [7]. Microscopy-based diagnostic tests have value in infection diagnosis but suffer from low sensitivity, are cumbersome to use, require expertise to perform, and lack throughput capability [8]. Some reports have indicated the rise of false negatives when using microscopic examination in situations such as reduced egg production following anthelminthic drug treatment and light infection [8]. Generally, they may not be useful in detecting low level infections characteristic of disease elimination campaigns, for monitoring success or failure of chemotherapy; they are neither adaptable for use in field settings nor adaptable for use at the point of care. Serodiagnostic tests have rarely been used for routine detection of* Ascaris* infections because they are invasive, detection of antibodies may persist after cure, and cross-reactions may also occur with other medical conditions [9]. Reliable diagnostic tests are therefore needed for infection diagnosis, monitoring of effectiveness of chemotherapy, and disease surveillance after elimination [10]. Molecular diagnostic tool, such as loop mediated isothermal amplification (LAMP), is a powerful alternative to conventional parasitological tests.

LAMP is a DNA amplification technology first described by Notomi and colleagues [11] but has now found wide application as a diagnostic tool for a wide variety of human, animal, and plant diseases caused by protozoans, bacteria, or viruses [12–16]. LAMP is capable of rapidly amplifying target nucleic acids with greater sensitivity, specificity, efficiency, and rapidity under isothermal conditions [16], without a significant influence of the copresence of nontarget DNA [17]. Furthermore, LAMP-based assays require relatively simple and inexpensive equipment such as an incubator, water bath, and heat block which can provide constant temperature and, therefore, can easily be applied at the point of care, even under field conditions [11, 16]. Recent studies have demonstrated that LAMP is more sensitive and specific than conventional polymerase chain reaction [18, 19]. Additional benefits include speed of the assay, shorter run time, ease of training personnel lacking a background in molecular techniques, and throughput capability. Thus, in this study, we developed a loop mediated isothermal amplification (LAMP) method for diagnosis of* Ascaris* infection in fecal samples.

## 2. Materials and Methods 

### 2.1. Specimen Collection and Preparation

#### 2.1.1. Parasite Material

A single adult* A. lumbricoides* specimen and parasite ova were used as sources of DNA for optimizing the LAMP assay. The adult worm was recovered from a fecal sample of a child who had received 400 mg albendazole [20], the standard dose for treatment of ascariasis. The specimen was preserved in 95% ethanol until used for DNA extraction and then discarded. Eggs of* A. lumbricoides* were obtained from fecal samples of infected school children, aged 6–12 years, from Msambweni subcounty in Kwale County, Kenya, whose parents had consented to their participation in this study. Ova of hookworm and* T. trichiura* were also isolated from the fecal samples of participating children, which provided DNA to verify specificity of the LAMP assay. In addition, ova of* S. mansoni* were also recovered from lab infected mice and these provided DNA for specificity testing of the assay. The* S. mansoni* ova were donated from an ongoing approved study within the institute.

#### 2.1.2. Fecal Sample Collection and Parasitological Examination

Each participating child provided a single fecal sample in a capped plastic polypot, which were transported back to the field laboratory in an ice box within 2 h of collection. Duplicate Kato-Katz microscope slides were prepared as per the Kato-Katz procedure [7] and examined under a compound microscope at ×400 magnification by two experienced microscopists. Additionally, 5% of the total number of slides was read by a third microscopist for quality control purposes.* A. lumbricoides* ova positive samples were used to isolate ova for DNA extraction used for optimizing the LAMP technique.

### 2.2. Isolation of* A. lumbricoides* Ova

Ova of* A. lumbricoides* were isolated from individual fecal samples using the modified Wisconsin's sugar flotation method [21]. Briefly, Sheather's sugar solution with a specific gravity of 1.27 was prepared by mixing 455 gm table sugar with 355 mL distilled water and boiled to dissolve the sugar. Approximately 6 gm of fecal sample was then placed in a 3.5-inch diameter petri dish and 30 mL of the Sheather's solution was added. The mixture was stirred into a homogenate using a wooden tongue depressor and filtered through a layered wet cheese cloth into a 50 mL centrifuge tube. The filtrate was centrifuged at 3000 rpm for 10 min, and the solution was left to stand for 10 min to allow the parasite eggs to float. The presence of parasite ova was confirmed under the microscope by placing a cover slip over the solution and transferring it onto a microscope slide [21]. Approximately 5 mL of the supernatant was transferred into a 15 mL centrifuge tube, and 2 volumes of water were added to dilute the sugar solution, consequently, reducing the specific gravity. This allowed the eggs to settle at the bottom after a 3 min spin in the centrifuge at 3000 rpm. The resulting pellet was resuspended in 2 mL distilled water, mixed by vortexing, and then transferred into 1.5 mL centrifuge tubes.

### 2.3. Designing LAMP Primers

Primer Explorer V4 (Fujitsu Ltd., Tokyo, Japan) was used for primer design [22]. Several primer sets were generated, and computation analysis of primers by the basic local alignment search tool (BLAST) (https://blast.ncbi.nlm.nih.gov/Blast.cgi) was performed to verify their suitability. Those that generated close relatedness to* A. lumbricoides*, from GenBank with sequence accession number AJ000895, were chosen for subsequent experiments. After several trials, only one set of primers from the ITS-1 was chosen having FIP, BIP, F3, and B3 (see Table 1 for the base sequences of the primers used in this study).

### 2.4. DNA Extraction

DNA was extracted from intact eggs of* A. lumbricoides*, as well as from an adult worm using the modified HotShot method [23]. Briefly, 1, 5, 10, 15, 20, and 25* Ascaris* eggs were placed into 6 separate PCR tubes, respectively, each containing 30 *µ*L of lysis reagent (NaOH, EDTA, and distilled water), and incubated at 95°C for 1 h. An equal volume of neutralizing reagent (TrisHCl and distilled water) was added to each of the tubes at the end of the incubation period [23]. The DNA concentration was determined for each tube to estimate the amount of DNA available for the different egg counts using the NanoDrop 2000 (Thermo Scientific, Wilmington DE, USA). To extract DNA from an ethanol-preserved adult* A. lumbricoides* worm, the specimen was cut up into tiny pieces, each measuring ~1 mm. These were then placed in a petri dish containing distilled water and soaked for 1 h to remove ethanol traces. Each piece was then transferred into a 200 *µ*L tube containing 40 *µ*L of lysis reagent and incubated at 95°C for 1 h and followed by addition of equal volume of neutralizing reagent (TrisHCl, distilled water) to end up with DNA solution in TE buffer [23]. To evaluate the specificity of the developed LAMP test, three other parasite ova were used, namely, hookworm,* Schistosoma mansoni*, and* Trichuris trichiura.* These are fecal-based helminth parasites that commonly coexist with* A. lumbricoides*. DNA from ova of* S. mansoni* and* T. trichiura* was extracted as described above using the HotShot method [23]. However, DNA from the hookworm samples was extracted using the QIAmp Fast DNA stool minikit (QIAGEN, Hilden, Germany).

### 2.5. Optimization of the LAMP Reaction Conditions

We experimented with several sets of ITS-1 LAMP-based primers. First, we assayed different ratios of the inner to outer primers in the following ratios: 2 : 1; 4 : 1; 6 : 1; and 8 : 1 [11–13, 15–18]. Secondly, several incubation time ranges between 15 min–2 h with intervals of 15 min were tested for a constant amount of DNA concentration and fixed primer ration. Thirdly, the ideal incubation temperatures for each incubation time were optimized. The temperatures tested ranged from 56°C to 63°C based on the base composition of each primer and the recommended melting temperature (*T*_m_) 56°C of each primer [11–13, 15–18]. Finally, two DNA polymerase enzymes, namely,* Bacillus stearothermophilus (Bst)* [11] and* Bacillus smithii (Bsm)* [24], were tested to determine which between the two had the optimum yield, while other reaction conditions were being held constant. From the results of these experiments, one primer set was selected due to its ability and consistency to readily amplify* Ascaris* DNA in the LAMP reaction (see Table 1).

### 2.6. The LAMP Assay

All reactions were incubated in a thermal cycler, GeneAmp PCR System 97 (Applied Biosystems, Singapore). After several optimization reactions, we finally settled on the following LAMP reaction conditions in a final reaction volume of 21 *µ*L with the following components: 1 *µ*L of each of the four primers (FIP, BIP, B3, and F3) in a ratio of 6 : 1 inner to outer primers; 8 U/*µ*L of* Bsm* DNA polymerase (Thermo Scientific); 10x* Bsm* buffer; 1.2x of 2x LAMP reaction buffer [1 M TrisHCl, KCl, MgSO_4_, (NH_4_)_2_SO_4_, Tween 20, Betaine, and dNTPs] [24]; and 1 *µ*L target DNA. Amplification was carried out at 63°C for 45 min, and* Bsm* DNA polymerase activity stopped by a final incubation at 80°C for 10 min.

### 2.7. Detection of LAMP Products

To detect amplicons, 5 *µ*L of the products was separated using 2% TAE agarose gel stained with SYBR Green safe DNA gel stain [11]. Also, visual detection of the amplicon was carried out using SYBR Green 1 nucleic acid gel stain 10,000 (Life Technologies) [17] diluted in a 1 : 10 ratio, and 1 *µ*L of the diluted dye was added to each amplification reaction for visual detection with the naked eye. Color change was observed and photographed using the Nikon Coolpix 4500.

### 2.8. Evaluation of the LAMP Assay Using DNA Extracted Directly from Fecal Samples in Comparison with Kato-Katz Technique

DNA was extracted from 40 fecal samples using the QIAamp Fast DNA stool minikit (QIAGEN, Hilden, Germany), as per the manufacturer's instructions. Briefly, 200 mg of stool was weighed and placed into a 2 mL tube. 1 mL of InhibitEX buffer was added and vortexed for 1 minute. The mixture was heated for 10 min at 95°C followed by vortexing for 15 s. The sample was centrifuged for 1 min at 14,000 rpm (full speed). In a 1.5 mL microcentrifuge tube, 15 *µ*L of proteinase K was placed and 200 *µ*L of the supernatant was added, followed by addition of 200 *µ*L buffer AL. the mixture was vortexed for 15 s. To the lysate, 200 *µ*L of absolute ethanol was added and vortexed. To QIAmp spin column, 600 *µ*L of lysate was added and centrifuged full speed. The spin column was placed into a new 2 mL collection tube and 500 *µ*L buffer AW1 was added and followed by centrifugation at full speed for 1 min. After placing the spin column in another collection tube, 500 *µ*L buffer AW2 was applied and spun for 3 min at full speed. The spin column was transferred to a fresh collection tube and span for another 3 min at full speed. The spin column was placed in a 1.5 mL microcentrifuge tube, and 200 *µ*L of ATE buffer was added, followed by incubation at room temperature for 1 min. It was then centrifuged at full speed to elute DNA. Samples were blinded as they had earlier been screened using Kato-Katz procedure. A contingency table was used to determine the percent agreement between the developed assay and Kato-Katz technique.

## 3. Results

### 3.1. Fecal Sample Collection and Parasitological Examination

Fecal samples were collected from a total 581 children and examined for intestinal helminth infections. Overall prevalence of the three intestinal helminth infections among the children examined was 40%, with prevalence of* Ascaris* infection being 7.9%.

### 3.2. Parasite Ova Isolation and DNA Extraction

The ova of* A. lumbricoides* were successfully isolated from positive fecal samples using the modified Wisconsin flotation method resulting in a relatively clean sample free of fecal debris. Intact* Ascaris* eggs provided sufficient DNA for LAMP amplification. There was a general trend of increase in DNA concentration with increase in egg count in a sample.  Table 2 shows DNA concentrations for the various egg counts.

### 3.3. Optimal Conditions

**Figure 1:**
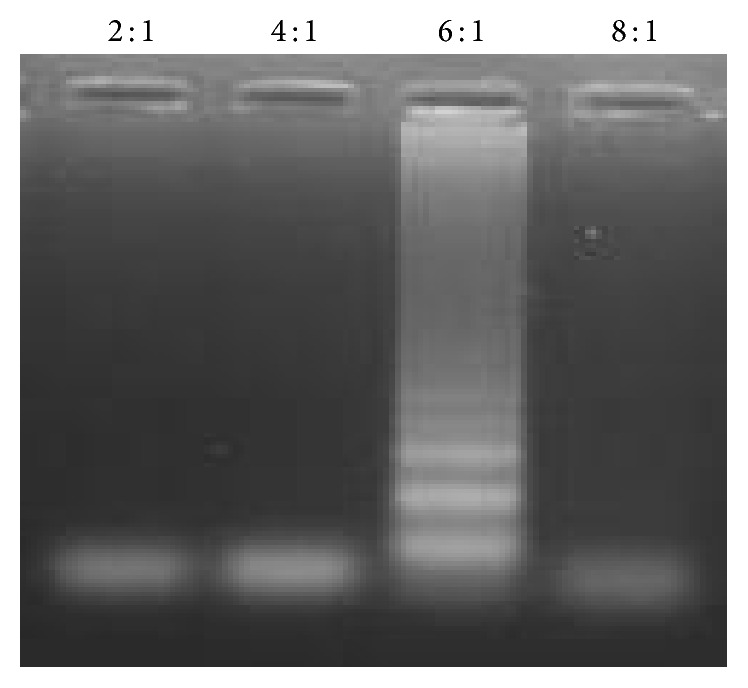
The various ratio of inner : outer primers tested. The 6 : 1 ratio readily amplified DNA of adult* A. lumbricoides* worm (positive control).

The optimum LAMP assay conditions were a temperature of 63°C for an incubation period of 45 min. The ideal ratio of inner to outer primers was found to be 6 : 1 (Figure 1).* Bsm* DNA polymerase was used instead of* Bst* DNA polymerase, as the latter consistently failed to produce any results under the optimized conditions.

### 3.4. Sensitivity of LAMP

**Figure 2:**
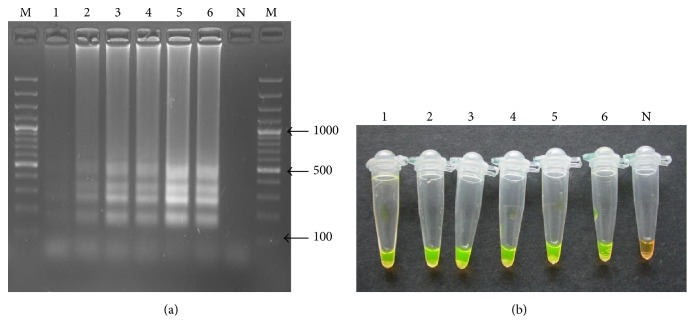
(a) Shows the sensitivity of LAMP visualized by 2% agarose gel electrophoresis. Lanes 1, 2, 3, 4, 5, and 6 are DNA extracted from 1, 5, 10, 15, 20, and 25 eggs, respectively. Lane M is the 100 bp molecular marker and N is the negative control. (b) LAMP visual detection for color change using the SYBR Green dye. Tubes 1, 2, 3, 4, 5, and 6 represent DNA extracted from 1, 5, 10, 15, 20, and 25 eggs, respectively, through 6. N is negative control.

The LAMP assay was able to detect DNA extracted from a single egg (DNA concentration 10.8 ng/*µ*L). This was confirmed by both gel electrophoresis and SYBR Green dye (Figures 2(a) and 2(b)).

### 3.5. Specificity of LAMP

**Figure 3:**
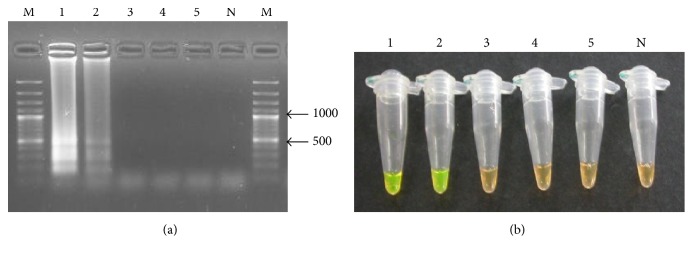
(a) Specificity of LAMP assay by gel electrophoresis. Lane 1,* A. lumbricoides* adult worm; lane 2,* Ascaris* ova DNA; lane 3, hookworm ova DNA; lane 4,* S. mansoni* ova DNA; lane 5,* T. trichiura* ova DNA; lane N, negative control; and M = 100 bp molecular ruler (Thermo Scientific). (b) Visual detection by color change using the SYBR Green dye. Tube 1,* A. lumbricoides* adult worm; tube 2,* Ascaris* ova DNA; tube 3, hookworm ova DNA; tube 4,* S. mansoni* ova DNA; tube 5,* T. trichiura* ova DNA; and lane N, negative control.

When* A. lumbricoides* specific primers were used to amplify DNA from hookworm and* T. trichiura* nematodes and* S. mansoni* trematode, which commonly coexist in feces with* Ascaris*, no cross-reactivity or amplification was observed, suggesting that the LAMP reaction was specific to* A. lumbricoides* (Figure 3).

### 3.6. Comparison of LAMP to Kato-Katz Technique

The developed LAMP technique was compared to Kato-Katz technique and results were displayed in 2 × 2 contingency tables (Tables 3 and 4), with sensitivity, specificity, and predictive values indicated.

## 4. Discussion

The loop mediated isothermal amplification (LAMP) test that we have developed is suitable for use in supporting ascariasis control efforts as it is able to detect low amount of parasite DNA when compared with Kato-Katz technique. The fact that the developed assay could detect parasite DNA from a single egg suggests that even low intensity* A. lumbricoides* infections can readily be detected in this assay. It has high throughput; therefore, many samples can potentially be handled at the same time.

The generated primers based on the ITS-1 region of the ribosomal DNA have been documented to be slow at accumulating substitutions and having lower intraspecific polymorphism [21]. In addition, organisms of the same genus have often been distinguished by ITS regions as they have higher sequence variability as compared to ribosomal DNA subunits, hence enhancing the potential to differentiate between* A. lumbricoides* and* A. suum*, which are closely related [25]. As a consequence, the developed LAMP assay did not cross-react with coexisting helminth parasites: hookworm,* T. trichiura*, or* S. mansoni*. This is important since in many endemic localities, multiple helminth parasite infections frequently occur in an individual.

One advantage of the LAMP test over conventional molecular techniques is that sophisticated equipment was not required for confirming the test results. For instance, a simple color change detection system based on staining the amplification products with SYBR Green dye was visualized with the naked eye, demonstrating the feasibility of using this assay under field conditions. This eliminates the need for gel electrophoresis. We also confirmed the amplicon products using gel electrophoresis, and the results were consistent with that of SYBR Green staining. In addition, since the assay reaction is isothermal, a simple water bath can be used for this test, potentially allowing the technique to be used at the point of care in low resource disease endemic regions. The DNA prepared by the method described by Truent and colleagues [23] contains impurities, yet the LAMP technology was able to reliably amplify target DNA, without significant influence of the copresence of nontarget DNA [17] and other impurities.

To validate the developed LAMP assay, 40 fecal specimens were used and when compared with Kato-Katz technique, which is the WHO gold standard [7], 85% agreement was present, 83.9% positive predictive value and 88.9% negative predictive value. Intermediate kappa of 0.72 was established [26]. The fact that LAMP could detect DNA from a single parasite ovum helps to illustrate the potential that the developed assay has for use in low infection set-up characteristic of disease control/elimination situation. Nonetheless, this study had a few limitations. The developed LAMP technique has reduced sensitivity when compared to Kato-Katz technique. This could be attributed to insufficient disruption of* Ascaris* ova which tend to have multiple proteinous layers, presence of natural DNA polymerase inhibitors in fecal samples [27], and probable degradation of DNA during storage. We microscopically confirmed insufficient ova disruption by observing drops of pellets from the first step of DNA extraction using QIAamp Fast DNA stool minikit. Numerous intact* Ascaris* ova could be seen in the samples.

## 5. Conclusion

These limitations notwithstanding, the LAMP assay developed for detection of* A. lumbricoides* infection in fecal samples is promising and could be useful as a diagnostic tool to support current ongoing efforts of ascariasis control in resource limited settings and in the evaluation of effectiveness of chemotherapy-based interventions. Furthermore it can be used in ascariasis diagnosis at the point of care and in low infection intensity situations, which are targeted in control and elimination campaigns. It is also a potentially useful tool for disease epidemiological surveillance and in operational research.

## Figures and Tables

**Table 1:** Set of primers used for amplification of *Ascaris lumbricoides* worm (positive control) and ova.

Primer	Sequence, 5′→3′
F3	CTTGTTAGAAAGGCATGCTAG
B3	GTGTTTTTTGAGTTTTGGCG
FIP	TAGCTCGGTGAAGCGTAGAC-**TTTT**-CTTATTTTCCCGCTATTTCGTA
BIP	GACCGTCGGTAGCGATGAAA-**TTTT**-GCTCATTGAGTCTACTCGA

**Table 2:** The concentration of DNA quantified using NanoDrop 2000 (Thermo Scientific) obtained for varying amounts of parasite ova.

No. of *Ascaris* ova from which DNA was extracted	Parasite ova DNA concentration in ng/*µ*L
1	10.8
5	13.1
10	19
15	30.5
20	38.1
25	44.2

**Table 3:** Contingency table assessing accuracy of LAMP in relation to Kato-Katz technique.

	Kato-Katz (gold standard)		Total
	Positive	Negative	
*LAMP*			
Positive	26	5	**31**
Negative	1	8	**9**
Total	**27**	**13**	**40**

Sensitivity = 26/27 × 100% = 96.3%.

Specificity = 8/13 × 100% = 61.5%.

Positive predictive value = 26/31 × 100% = 83.9%.

Negative predictive value = 8/9 × 100% = 88.9%.

**Table 4:** Agreement expected by chance alone of LAMP and Kato-Katz technique.

	Kato-Katz (gold standard)		Total
	Positive	Negative	
*LAMP*			
Positive	18.2	12.8	**31**
Negative	8.8	0.2	**9**
Total	**27**	**13**	**40**

Percent agreement expected by chance alone = (18.2 + 0.2)/40 × 100 = 46%.

Kappa = ((percent agreement observed) − (percent agreement expected by chance alone))/(100%  − (percent agreement expected by chance alone)) = (85 − 46)%/(100 − 46)% = 0.72%.

## Additional Points

All the children found to be infected with* A. lumbricoides* or other soil-transmitted helminths received the WHO recommended standard treatment for STHs of 400 mg albendazole [20] in a single dose under the supervision of a qualified clinician. Fecal sample collection for STH infection diagnosis is a routine procedure for parasitological investigations and does not pose any risks or cause any harm to participating children.
